# An improved method for the visualization of conductive vessels in *Arabidopsis thaliana* inflorescence stems

**DOI:** 10.3389/fpls.2015.00211

**Published:** 2015-04-09

**Authors:** Radek Jupa, Vojtěch Didi, Jan Hejátko, Vít Gloser

**Affiliations:** ^1^Department of Experimental Biology, Faculty of Science, Masaryk UniversityBrno, Czech Republic; ^2^Functional Genomics and Proteomics of Plants, Central European Institute of Technology, Masaryk UniversityBrno, Czech Republic

**Keywords:** conductive elements, dye perfusion, fluorescence, Fluorescent Brightener 28, hydraulic conductivity, vessel, xylem

## Abstract

Dye perfusion is commonly used for the identification of conductive elements important for the study of xylem development as well as precise hydraulic estimations. The tiny size of inflorescence stems, the small amount of vessels in close arrangement, and high hydraulic resistivity delimit the use of the method for quantification of the water conductivity of *Arabidopsis thaliana*, one of the recently most extensively used plant models. Here, we present an extensive adjustment to the method in order to reliably identify individual functional (conductive) vessels. Segments of inflorescence stems were sealed in silicone tubes to prevent damage and perfused with a dye solution. Our results showed that dyes often used for staining functional xylem elements (safranin, fuchsine, toluidine blue) failed with *Arabidopsis*. In contrast, Fluorescent Brightener 28 dye solution perfused through segments stained secondary cell walls of functional vessels, which were clearly distinguishable in native cross sections. When compared to identification based on the degree of development of secondary cell walls, identification with the help of dye perfusion revealed a significantly lower number of functional vessels and values of theoretical hydraulic conductivity. We found that lignified but not yet functional vessels form a substantial portion of the xylem in apical and basal segments of *Arabidopsis* and, thus, significantly affect the analyzed functional parameters of xylem. The presented methodology enables reliable identification of individual functional vessels, allowing thus estimations of hydraulic conductivities to be improved, size distributions and vessel diameters to be refined, and data variability generally to be reduced.

## Introduction

Recently, research on transport tissues (xylem, phloem) in higher plants has been attracting more attention (Wheeler and Stroock, 2008; Choat et al., 2012). Xylem, as a specialized long-distance transport tissue connecting roots with upper plant parts, typically consists of parenchyma cells, sclerenchyma fibers, and conductive elements. There are two basic types of conductive elements—tracheary elements (forming vessels) and tracheids. The conductive elements are elongated in the apical/basal axis and connected by microstructures called pits (Tyree and Zimmermann, 2002). The conductive elements are unable to transport water through their lumen until their development has been completed by the programmed death of protoplast followed by the partial hydrolysis of non-lignified primary cell walls (Bollhöner et al., 2012). Embolism, tyloses, or mechanical damage can turn functional conductive elements (able to transport water) into non-functional ones (Hacke and Sperry, 2001; Tyree and Zimmermann, 2002; Sun et al., 2006; Zhao et al., 2014). Although non-conductive elements represent a significant proportion of xylem (Rančić et al., 2008; Halis et al., 2012), little attention has been paid to their identification in analyses of xylem functioning.

In several studies, the histochemical staining of cross sections by toluidine blue or acidified phloroglucinol was used for the identification of conductive elements with lignified secondary cell walls (Chaffey et al., 2002; Jupa et al., 2013). However, the degree of lignification is not a reliable marker for the identification of fully functional elements. In contrast to these methods, the perfusion of a tracer (a dye solution with the ability to adhere to components of secondary cell walls in particular) through conductive pathways is frequently utilized to identify functional elements (Sano et al., 2005; Umebayashi et al., 2007; Halis et al., 2012). Such a method can be useful not only in the study of xylem development but also offers significant benefits in the evaluation of hydraulic properties. Over the past 25 years, the dye perfusion method was mostly used for the visualization of conductive pathways in trees, shrubs or lianas, while studies on herbaceous plant species were less frequent (Supplemental Table 1).

*Arabidopsis thaliana* is one of the model organisms most frequently used in recent plant research (Hejátko et al., 2009; Zhang et al., 2011; Lee et al., 2013). Differences in functional anatomy and ontogenetic xylem development among mutant lines have attracted attention in several areas of plant science (Nieminen et al., 2004). Moreover, a recent study by Tixier et al. (2013) proposes *Arabidopsis* as a model species for the study of xylem hydraulics. Although a technique for the precise visualization of functional vessels in *Arabidopsis* plants would be beneficial in similar studies, none has yet been developed, probably because of several methodological limitations typically relating to this plant species, such as the high risk of mechanical damage during manipulation of the tiny and fragile inflorescence stems, the high resistance of the conductive pathway, the progressive diffusion rate of a dye solution in herbaceous stems, the small number of vessels closely arranged in collateral vascular bundles, and (in particular) the difficulties in distinguishing vessels from surrounding cells in cross sections.

Such methodological limitations require improvement of the dye perfusion technique for *Arabidopsis thaliana* plants. In this paper, we introduce an optimized technique based on perfusion with a fluorescent dye solution which enables the reliable identification of individual conductive vessels in inflorescence stems of *Arabidopsis thaliana*. The results of our anatomical analysis of xylem vessels and hydraulic measurements demonstrate the importance of accurate identification of functional vessels.

## Materials and methods

### Plant material

Seeds of wild-type (WT) *Arabidopsis thaliana* (L.) Heyhn. plants of the Columbia ecotype (Col-0) were sown on Petri dishes with modified Murashige-Skoog medium supplemented by 1% agar and 1% sucrose. Plants grew in an environmentally controlled room under white light (with an irradiance of 150 μmol m^−2^ s^−1^) with a 16/8 h light/dark cycle and a relative humidity of 60% at 19/21°C for 14 days. The plants were then replanted into a mixture of soil (TS-3; Klasmann-Deilmann, Geeste, Germany), perlite, and sand in the ratio of 12:3:4, respectively, and grown in identical conditions until the first siliqua ripening developmental period was reached (~4 weeks). The plants were then used without delay in dye perfusion experiments.

### Stem segment adjustment

Typical subtle inflorescence stem segments were adjusted to undergo hydraulic measurements and the dye perfusion described below. The main inflorescence stem was completely defoliated and disbranched with a sharp razor blade to stop transpiration and reduce embolism. It was then cut off right above the leaf rosette. The remains of leaves and axillary branches were sealed with glue (Super Bond 409; Loctite Corporation, Rocky Hill, CT, USA). Two stem segments (from the apical and basal parts) were prepared from each inflorescence stem. The apical segment was cut off by razor blade at a distance of 20 mm in the basipetal and acropetal direction from the last lateral branch. The basal stem segment was cut off at a distance of 40 mm from the basal end of the inflorescence stem. Only stem segments with one lateral branch junction were used for the subsequent processing; other segments were discarded. This prevented discrepancies in the measurement of experimental hydraulic conductivity (*K*_h_) due to a different number of junctions and also reduced the staining of parenchyma cells in the pith region. Each stem segment was placed into a 5 cm long silicone tube of internal diameter 3 mm. The lumen of the tube with the inserted stem segment was completely filled with a condensation curing silicone impression material of low viscosity (Siloflex Plus Light; SpofaDental, Jičín, Czech Republic) mixed with a paste catalyst in the ratio of ~10:1. The mixture was drawn into the tube by means of a syringe connected to its basal end. After the stiffening of the Siloflex mixture (15–20 min), the silicone tube was trimmed without delay to a final length of 30 mm by cutting off 5 mm overlaps of the stem segment at both ends in water.

### Hydraulic measurement

Immediately after shortening, the 30 mm long silicone tube containing the sealed stem segment was connected to an apparatus to measure flow rate and acropetally flushed with a filtered (0.2 μm), degassed 10 mM KCl solution at a pressure of 100 kPa for 20 min to remove embolism. The connections formed with the silicone tube end-pieces were tightened by gluing plasticine (UHU Patafix; UHU Ltd., Bühl, Germany) and Parafilm (Pechiney Plastic Packaging Company, Chicago, IL, USA) around them. After embolism removal, the flow rate was measured for 30 min under a stable pressure gradient of 40 kPa by directing the solution to a precise analytical balance (Mettler-Tolledo XS205; Mettler-Toledo, Giessen, Germany) connected to a computer with LabView 8.0 software (National Instruments, Austin, TX, USA) to acquire the data in 20 s intervals (Sperry et al., 2005). The experimentally measured hydraulic conductivity (*K*_h_) was calculated as:

$$K_{h}=\frac{FL}{\Delta P}$$

Where *F* is the flow rate, *L* is the length of segment, and Δ*P* is the pressure gradient.

### Dye perfusion

Dye perfusion was performed immediately after the measurement of conductivity. The silicone tube segment was disconnected from the apparatus for measuring flow rate and the silicone end-pieces filled with a small amount of KCl solution to prevent embolism introduction. The silicone tube segment was then connected by its apical end-piece to a membrane vacuum pump with regulated suction (KNF N816; KNF Neuberger, Freiburg im Breisgau, Germany). The basal end of the silicone tube segment was immediately submerged into a cuvette placed on the precise analytical balance and filled with aqueous 0.3% (w/w), filtered (0.2 μm), degassed Fluorescent Brightener 28 (FB28) dye solution (Sigma-Aldrich, St Louis, MO, USA, molecular weight = 916.98 g mol^−1^, excitation 395–415 nm and emission 455 nm maxima; Garcia, 2002). The FB28 dye is an optical brightening agent commonly used in cell biology to stain cell walls and other structures containing cellulose or chitin. It cannot penetrate intact cell membranes at the same time and thus does not stain living cells (Mason et al., 1995). Selected stem segments were perfused with aqueous solutions of safranin T, toluidine blue, and acid and basic fuchsine (Sigma-Aldrich, St Louis, MO, USA) at concentrations of 0.05, 0.5, 0.2, and 0.2% (w/w), respectively. The silicone tube segment was fixed in a stable position to prevent contact with the cuvette walls. The dye solution was then acropetally drawn into the stem segment at a maximal pressure of −80 kPa generated by the vacuum pump. The stable rate of pulling and the amount of pulled dye solution were determined online from the solution weight decrease. The mass of the perfused dye solution (basal segment, ~0.1 g; apical segment, 0.05 g) was proportional to the size of the stem segment and simultaneously to the volume of xylem vessels. After dye application, filtered (0.2 μm) and degassed distilled water was perfused by the same technique to remove redundant dye from xylem vessel lumina. The amount of perfused water was approximately two times greater than the amount of drawn dye solution.

### Preparation of cross sections and microscopic observations

Immediately after water perfusion, the silicone tube segment was longitudinally incised and the sealed stem segment was removed. Subsequently, 50 μm-thick native cross sections were prepared by means of a vibrating blade microtome (Leica VT1200 S; Leica Microsystems Ltd., Wetzlar, Germany) at a distance of 5 mm from the outflow end and then mounted in water. Several cross sections were stained with a 0.05% (w/v) solution of toluidine blue in water for 1 min and destained in distilled water for 30 s. The native cross sections were observed using both a bright field (BF) microscope and an epifluorescence (EF) microscope (Olympus BX61; Olympus Corp., Tokyo, Japan; filter set U-MNUA2) equipped with a mercury lamp. Two analogous pictures (BF and EF) of each individual vascular bundle were photographed with a digital camera (Olympus DP70; Olympus Corp., Tokyo, Japan) at 40× magnification. In addition, some of the selected cross sections were observed with an Olympus IX81 FluoView 500 confocal laser scanning microscope (CLSM) (Olympus Corp., Tokyo, Japan) at 40× magnification. The optical thickness of slices was 2.53 μm. The excitation wavelength was 405 nm. The proper setting of the exposure time in the EF observation was necessary to obtain an image suitable for the subsequent analysis. The exposure time was set to reduce undesirable fluorescence signals. The most intense signal from stained secondary cell walls of xylem vessels was obtained with an exposure time of between 100 and 500 μs without any autofluorescence signal. The possible slight staining of surrounding tissues in the protoxylem (PX) area was reduced by setting the exposure very finely or, additionally, during image analysis processing (Figure not shown).

### Data analysis

**Figure 1:**
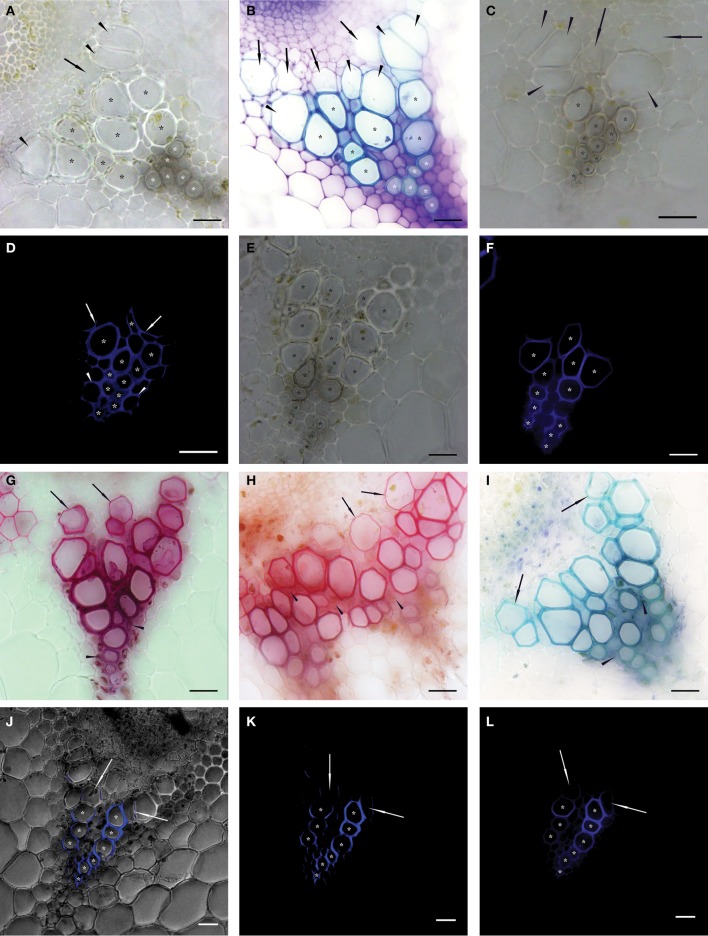
**Native cross sections of individual vascular bundles in inflorescence stems of *Arabidopsis thaliana***. Asterisks show vessels identified as functional (conductive) in 50 μm thick transverse sections observed in bright field microscopy—BF **(A–C,E)**; epifluorescence microscopy—EF **(D,F,L)**; and confocal scanning laser microscopy—CLSM **(J,K)**. Vessels were identified as functional if their secondary cell wall was fully developed (BF) or more than one half of their perimeter was stained after proper setting of the exposure time (EF and CLSM). **(A)** Transverse section of non-stained vascular bundle. White asterisks mark protoxylem (PX) vessels and black asterisks mark metaxylem (MX) vessels identified as conductive. Arrowheads show expanded but not yet lignified MX vessels, arrows show expanding vessel cells **(A–C)**. **(B)** Transverse section histochemically stained with toluidine blue prepared sequentially in a basipetal direction from the section in **(A)**. White asterisks mark PX vessels and black asterisks mark fully lignified MX vessels considered as conductive. **(C)** Cross section prepared from apical stem segment perfused with Fluorescent Brightener 28 (FB28) dye solution. **(D)** The identical cross section to **(C)**. Asterisks show stained secondary cell walls of conductive vessels perfused with the dye; non-lignified vessels are non-conductive and thus not stained; arrows show partially stained secondary cell walls of non-conductive MX vessels; arrowheads show stained fibers in PX area. **(E)** Cross section prepared from basal stem segment perfused with FB28 dye solution. **(F)** The identical cross section to **(E)**. Compared to **(E)**, seven fewer MX vessels were identified as conductive. **(G–I)** Images of vascular bundles in transverse sections prepared from basal segments perfused with basic fuchsine **(G)**, safranin **(H)**, and toluidine blue **(I)** solutions observed in BF. Arrows show stained but not yet lignified vessels; arrowheads show the intense staining of fibers. **(J–L)** An identical transverse section of a vascular bundle prepared from a basal segment perfused with FB28 dye solution and observed in CLSM **(J,K)** and in EF **(L)**. Asterisks show the analogical number of vessels considered as conductive; arrows show partially stained vessels considered as non-conductive. **(J)** Permeation of a BF image with the signal from the FB28 solution (shown in **K**) perfused through conductive vessels and excited at a wavelength of 405 nm. Scale bars 20 μm.

Vessels of protoxylem (PX), metaxylem (MX), and secondary xylem (SX) were defined according to Figures 1A,B. Due to the small number of SX vessels, they were included in the MX group. Image analysis was used for the determination of individual conductive vessels. Functional vessels were identified according to two approaches utilizing different observations of analogical cross sections. First, vessels with fully developed secondary cell walls were considered as conductive in BF pictures, except for expanding vessels (Figures 1C,E). Second, vessels perfused with FB28 dye solution in which more than one half of the secondary cell wall perimeter was stained after exposure setting were considered as conductive in EF pictures (Figures 1D,F,L; Supplemental Figures 1C,E). In anatomical analyses, conductive vessels were considered as ideal capillaries of circular cross section. The lumen area of each conductive vessel was precisely selected in Adobe Photoshop CS6 (Adobe Systems Inc., San Jose, CA, USA) and evaluated in ImageTool v. 3 software (Health Science Center, University of Texas, San Antonio, TX, USA), and the diameter was calculated. The total theoretical hydraulic conductivity (*K*_ht_) of the lumen was calculated as:

$$K_{ht}=\sum \frac{\pi D^{4}}{128\eta }$$

Where *D* is the vessel diameter and η is the dynamic viscosity of water at 20°C (1.002 × 10^−9^ MPa·s). The experimental specific conductivity (*K*_hs_) and theoretical specific conductivity (*K*_hts_) were calculated as the division of *K*_h_ or *K*_ht_ by the total area of the vessel lumen.

### Statistical analysis

The specific statistical approaches applied are indicated in the text. The results are presented in the form of mean ± standard error (number of replicates), unless stated otherwise. Statistica v. 12 software (StatSoft Inc., Tulsa, OK, USA) was used for all statistical analyses of the data.

## Results

### Experimental improvement of the methodology

Several methodological improvements were required to successfully conduct hydraulic measurements and the process of dye application. First, damage-free manipulation of the subtle inflorescence stems of *Arabidopsis* was necessary. To ensure this, we used silicone impression material to seal the analyzed stem segments in silicone tubes, which provided protection against mechanical damage and facilitated safe sample handling, important for the reduction of errors in measurements.

The length of the segment also had a significant effect on the results of staining, in particular. Short segments (<1 cm) were unusable because the dye solution was pulled into intercellular spaces under the generated tension, resulting in intense staining of the whole cross section in the outflow end, which complicated vessel identification. In contrast, the use of longer segments (3 cm) provided an advantage by reducing the staining of surrounding tissues. Besides this, discrepancies in hydraulic measurements were reduced by using segments with one branch junction, although no significant differences in resistivity between branch junctions and internode segments of the same length were shown (*P* = 0.287, paired t-test for dependent samples). The lumen resistivity of the junctions (2.02E+09 ± 8.00E+08 MPa·s·m^−4^, *n* = 8) was slightly higher than that of the internodes (7.93E+08 ± 5.19E+08 MPa·s·m^−4^, *n* = 8).

To ensure the prompt and uniform delivery of the dye solution into the fragments of silicone-sealed inflorescence stem, application by means of suction generated by a vacuum pump connected to the outflow end of the segment was found to be the most suitable. The preparation of native cross sections was performed using a vibratome (vibrating blade microtome), although the risk of diffusion was higher in comparison to that when using fixatives and a cryo-microtome. Nevertheless, this approach allowed prompt sectioning followed by immediate microscopy observation. No significant diffusion of dye was observed in cross sections up to 2 h after staining, thus reducing the number of false positives in the determination of functional vessels. As we discovered, dye photodecomposition by excitation irradiance (bleaching) caused more substantial discrepancies in the determination of vessels than diffusion of the dye, though Fluorescent Brightener 28 (FB28) was more resistant to bleaching than other fluorescent dyes (e g., Texas Red).

### The perfusion of inflorescence stems with fluorescent brightener 28 dye allowed the reliable identification of functional conductive tissue in *Arabidopsis* inflorescence stems

Before the testing of individual tracers suitable for the identification of conductive vessels, we investigated differences in the identification of functional vessels based on the development of their secondary cell walls observed in cross sections. There was no evident difference between non-stained native cross sections and cross sections histochemically stained with toluidine blue. Lignified fully-developed secondary cell walls of vessels which were considered to be functional in non-stained BF cross sections were stained by toluidine blue as well (Figures 1A,B; Supplemental Figures 1A,B).

The results of staining after the perfusion of segments with safranin, toluidine blue, and basic fuchsine solutions showed similarities. All the dyes always stained fibers in the area of PX (Figures 1G–I). Because of the similar staining intensities of fibers with PX vessels, the identification of their secondary cell walls was not possible. The staining of secondary cell walls with the greatest contrast was observed in MX vessels perfused with basic fuchsine and toluidine blue. Although the staining intensity decreased centrifugally in the direction of developing vessels, secondary cell walls of expanded but not yet lignified vessels were stained as well (Figures 1G,I). Safranin dye stained almost the whole vascular bundle (including vessels and fibers) as well as interfascicular arcs. Thus, it was impossible to identify individual functional vessels (Figure 1H). Secondary cell walls of vessels perfused with acid fuchsine were stained poorly or not at all, because the negatively charged dye was unable to adhere to the secondary cell wall components and was removed as a consequence of water perfusion.

In contrast, the identification of functional vessels in segments perfused with FB28 dye solution was much clearer. The visual identification of stained secondary cell walls of functional vessels was possible both in epifluorescence microscopy (EF) and confocal laser scanning microscopy (CLSM) images. Consequently, the numbers of cells identified as conductive vessels were identical in all compared images (Figures 1K,L). These images clearly show that the fluorescence signal from secondary cell walls with bound FB28 dye was most intensive compared to signals from other tissues. The autofluorescence signal from surrounding parenchyma cells was filtered out by means of proper exposure settings; the exposure time necessary to detect the autofluorescence signal from control non-perfused segments (100–200 ms) was at least three orders of magnitude longer compared to the exposure times needed to detect the signal from FB28 dye bound to the secondary cell walls of perfused vessels (100–500 μs).

Microscopic observations in EF and CLSM revealed the staining of fibers situated in close proximity to the protoxylem (PX) area, similar to perfusions with basic fuchsine and toluidine blue. Nevertheless, spiral secondary cell walls of stained PX vessels were observable in sufficient contrast. The fluorescence intensity from stained PX secondary cell walls was generally lower in comparison to metaxylem (MX) vessels (Supplemental Figures 1E,F). The secondary cell walls of MX vessels were stained without intensive staining of surrounding fibers. Secondary cell walls of non-conductive vessels were not stained at all or only up to one half of the perimeter length (vessels of MX or SX in contact with conductive vessels, Figure 1D; Supplemental Figure 1E). Because the application of FB28 dye showed the best staining results from all of the tested dyes, it was used for all subsequent analyses.

### Functional vessel identification substantially affects the determination of anatomical parameters

The identification of vessels using FB28 dye perfusion substantially influences the values of anatomical parameters. Paired *t*-test for dependent samples was used for testing differences between BF and EF in all following parameters, unless stated otherwise. We combined data sets from both the apical segments and the basal segments to obtain more robust validation of the method.

#### Characterization of functional vessels

The comparison of diameters calculated from the lumen of corresponding vessels observed in BF and EF images revealed statistically significant differences (*P* < 0.001, Wilcoxon test for paired samples). The mean diameter of vessels observed in BF was 9.43 ± 0.22 μm (*n* = 301), while the corresponding value for the identical vessels observed in EF was 9.66 ± 0.22 μm (*n* = 301).

Significant differences were also revealed in the numbers of conductive vessels identified from BF and EF images. In each stem segment, there were on average 30% fewer PX vessels (*P* < 0.001, Wilcoxon test for paired samples) in EF (43 ± 3, *n* = 22) when compared to BF (61 ± 3, *n* = 22). In parallel, there were on average 43% fewer MX and SX vessels (*P* < 0.001, Wilcoxon test for paired samples) in EF (23 ± 4, *n* = 22) when compared to BF (40 ± 7, *n* = 22). Thus, the difference in the total vessel count was 35% (*P* < 0.001) between EF (66 ± 5, *n* = 22) and BF (102 ± 9, *n* = 22). The number of non-functional vessels was proportional to the total vessel count, as confirmed by the significant correlation (Supplemental Figure 2, *P* < 0.001). The data achieved best fit with linear regression (*R*^2^ = 0.7489).

The difference between the mean diameter calculated from BF (10.30 ± 0.60 μm, *n* = 22) and that calculated from EF (10.49 ± 0.56 μm, *n* = 22) was not statistically significant (*p* = 0.369) for the apical segments analyzed together with the basal segments, but was statistically significant for separate apical segments (Table 1).

**Table 1:** **Comparison of inner vessel diameters—*D* (μm), theoretical hydraulic conductivity—*K*_ht_ (m^4^ MPa^−1^ s^−1^), experimental specific conductivity—*K*_hs_ (m^2^ s^−1^ MPa^−1^), and theoretical specific conductivity—*K*_hts_ (m^2^ s^−1^ MPa^−1^)**.

**Acronym**	**Apical (*n* = 11)**				***P***	**Basal (*n* = 11)**				***P***
	**BF**		**EF**			**BF**		**EF**		
	**Mean**	***SE***	**Mean**	***SE***		**Mean**	***SE***	**Mean**	***SE***	
*D*	7.68	0.31	8.26	0.25	0.002	12.92	0.26	12.73	0.49	0.598
*K*_ht_	1.69E-11	4.26E-12	1.13E-11	1.82E-12	0.016	2.19E-10	1.68E-11	1.11E-10	2.05E-11	<0.001
*K*_hs_	0.00095	0.00024	0.00118	0.00028	0.040	0.00155^*^	0.00024	0.00286^*^	0.00040	<0.001
*K*_hts_	0.00405	0.00054	0.00376	0.00036	0.210	0.01002^*^	0.00032	0.00810^*^	0.00052	0.021

Identification of conductive vessels was based on secondary cell wall development (BF) and the staining of secondary cell walls of vessels perfused with Fluorescent Brightener 28 dye solution (EF) in identical transverse sections from separate apical and basal segments. Symbol

* *represents n = 10*.

Theoretical hydraulic conductivity (*K*_ht_) represents a critical parameter in the functional characterization of vascular tissues. Similarly to the aforementioned number of vessels, differences in *K*_ht_ calculated from the diameters of stem segments identified in BF and EF cross sections were tested. Values of *K*_ht_ based on the BF analysis (1.18E-10 ± 2.36E-11 m^4^ MPa^−1^ s^−1^, *n* = 22) were significantly higher (*P* < 0.001, Wilcoxon test for paired samples) than *K*_ht_ calculated from EF (6.14E-11 ± 1.49E-11 m^4^ MPa^−1^ s^−1^, *n* = 22).

#### Comparison of hydraulic conductivities

**Figure 2:**
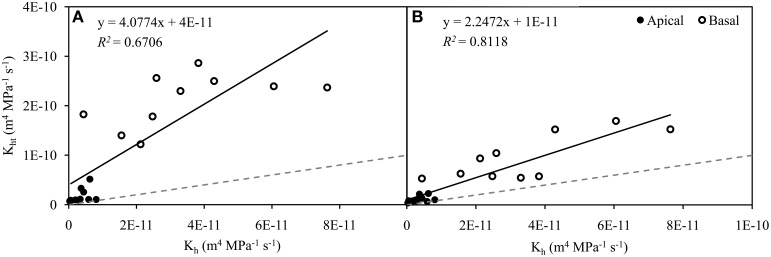
**Relationship between experimental hydraulic conductivity (*K*_h_) and theoretical hydraulic conductivity (*K*_ht_) in apical (*n* = 11, closed circles) and basal (*n* = 10, open circles) inflorescence stem segments of *Arabidopsis thaliana***. Theoretical hydraulic conductivity was calculated from the identification of conductive vessels, which was based on the development of secondary cell walls observed in bright field **(A)** and the staining of secondary cell walls perfused with Fluorescent Brightener 28 dye solution and observed in epifluorescence **(B)**. Transverse sections used in both identification approaches were identical. Data were significantly correlated (*P* < 0.001) and best fits were achieved with linear regressions (**A**, *R*^2^ = 0.6706; **B**, *R*^2^ = 0.8118). Gray dashed lines represent values in the ratio 1:1.

A strong linear correlation (*P* < 0.001) was found in the relationship between *K*_h_ and *K*_ht_ determined from BF (*R*^2^ = 0.6706). However, the correlation coefficient increased substantially (*R*^2^ = 0.8118) after the identification of functional vessels in EF images. In addition, the linear regression approached close to the ideal correlation between *K*_h_ and *K*_ht_ (1:1) after the identification of conductive vessels in EF (Figures 2A,B). This significant improvement in the quality of the relationship clearly shows the importance of the contribution of the identification method.

The relative ratio of *K*_h_ and *K*_ht_ calculated from BF and EF images showed a significant difference of 12.88% (*P* < 0.001, Wilcoxon test for paired samples). With respect to *K*_ht_ calculated from BF images, the relative contribution of *K*_h_ was 20.12 ± 3.75% (*n* = 22). After the identification of functional vessels by FB28 dye perfusion, the contribution of *K*_h_ related to *K*_ht_ increased to 33.00 ± 4.84% (*n* = 22). This difference in the discrepancy between *K*_h_ and *K*_ht_ is clearly due to the incorrect identification of functional vessels, particularly in BF images. Importantly, as a further consequence of the correct identification of functional vessels by dye perfusion, data variation was reduced. The coefficient of variation for the relative ratio of *K*_h_ and *K*_ht_ decreased from 0.87 to 0.69 when calculated from BF and EF, respectively.

There were also significant differences (*P* < 0.001) between *K*_hs_ determined from BF (0.0012 ± 0.0002 m^2^ s^−1^ MPa^−1^, *n* = 21) and EF (0.0020 ± 0.0003 m^2^ s^−1^ MPa^−1^, *n* = 21) images. Similarly, values of *K*_hts_ determined from BF (0.0069 ± 0.0007 m^2^ s^−1^ MPa^−1^, *n* = 21) were significantly higher (*P* = 0.011) than corresponding values from EF (0.0058 ± 0.0006 m^2^ s^−1^ MPa^−1^, *n* = 21).

**Figure 3:**
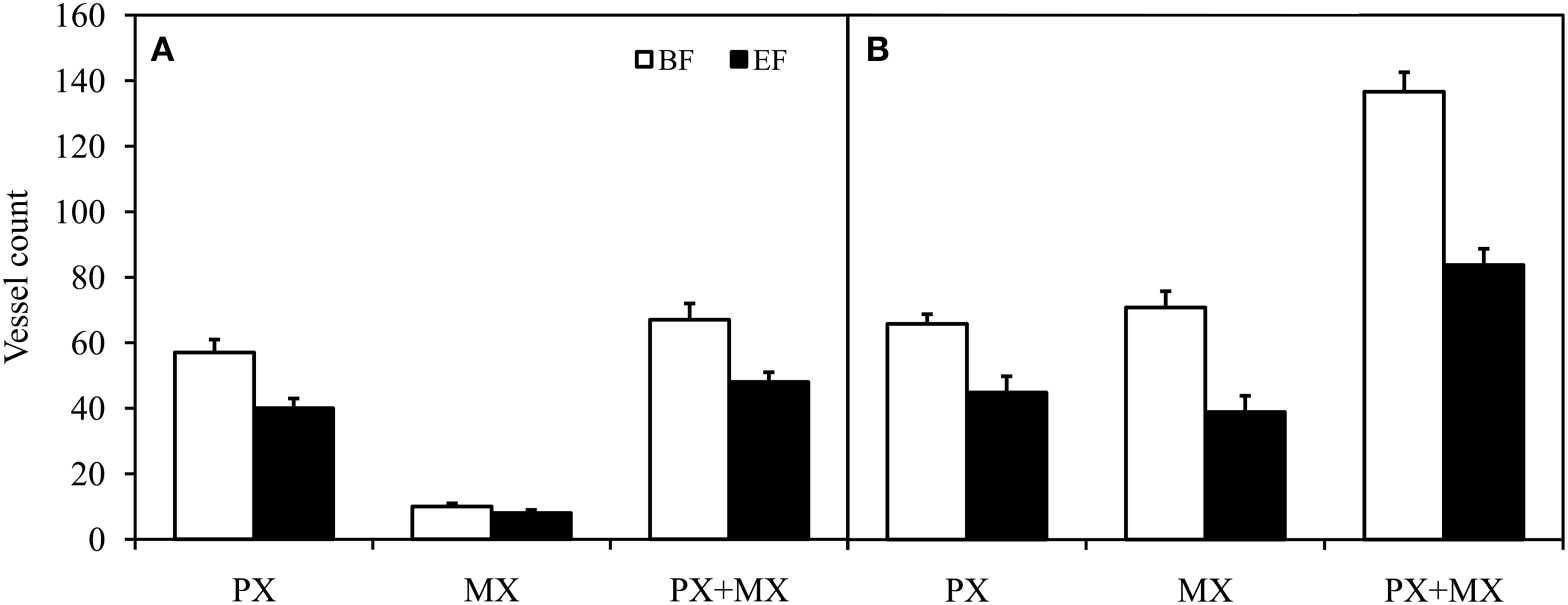
**Comparison of protoxylem (PX) conductive vessel count, metaxylem (MX) conductive vessel count, and total (PX + MX) conductive vessel count in apical (A) and basal (B) inflorescence stem segments of *Arabidopsis thaliana***. Conductive vessels were identified according to the development of secondary cell walls observed in bright field (BF) and the staining of secondary cell walls perfused with Fluorescent Brightener 28 dye solution and observed in epifluorescence (EF). There were statistically significant differences (*P* < 0.01) between conductive vessel identification methods in all groups of vessels. Means are given ± SE (*n* = 11).

**Figure 4:**
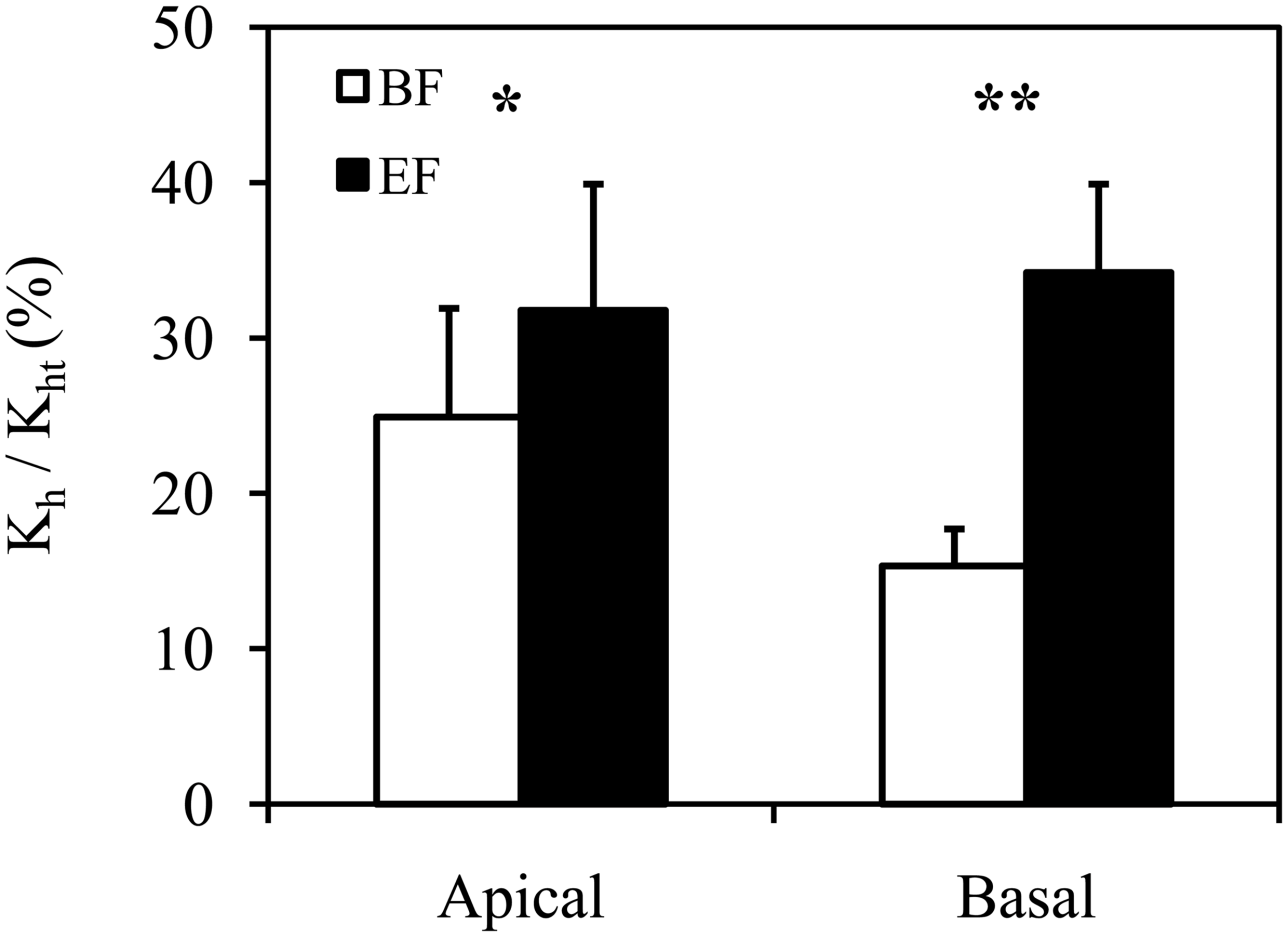
**Relative ratio of experimentally measured hydraulic conductivity (*K*_h_) and theoretical hydraulic conductivity (*K*_ht_) in apical and basal inflorescence stem segments of *Arabidopsis thaliana***. Conductive vessels were identified according to the development of secondary cell walls observed in bright field (BF) and the staining of secondary cell walls perfused with Fluorescent Brightener 28 dye solution and observed in epifluorescence (EF). Statistically significant differences are indicated as ^*^P < 0.05 and ^**^P < 0.01. Means are given ± SE (*n* = 11).

The anatomical measurements and hydraulic estimations described above, evaluated from cross sections observed in BF and EF, were also tested in apical and basal segments separately (Table 1; Figures 3, 4). Values of *K*_h_ were 3.45E-12 ± 7.71E-13 m^4^ MPa^−1^ s^−1^ (*n* = 11) in apical segments and 3.41E-11 ± 6.12E-12 m^4^ MPa^−1^ s^−1^ (*n* = 11) in basal segments. Significant differences were found for all aforementioned parameters, except *K*_ht_ in apical segments and mean diameter in basal segments.

## Discussion

In general, dye perfusion is a simple method providing generally good results with respect to the visualization of conductive elements. In many studies, dye perfusion techniques were used to visualize conductive pathways and connections in xylem structures as well as to identify the conductive parts of xylem in plant species which generally contained many conductive elements (Supplemental Table 1). In contrast, *Arabidopsis thaliana* inflorescence stems typically display small numbers of narrow vessels (Tixier et al., 2013). Hence, even small fluctuations in the determination of the number of functional vessels can substantially affect the results of studies on xylem functioning, thus further substantiating the need for precise identification of the functionality of individual vessels.

### Measurement of *K*_h_

The evaluation of *K*_h_ was important for the verification of the dye perfusion technique, and the complete removal of embolism from xylem vessels was an important first step in this measurement. The time needed for embolism removal varies among plant species. In contrast to Tixier et al. (2013), the time for safe embolism removal in *Arabidopsis* stems was four times longer in our study; this was for two main reasons. First, we did not cut the inflorescence stem under water because a thin water layer on the stem surface prevents the adherence of the silicone impression material, which can lead to leakage along the surface of the sealed segment. Second, we used longer segments exceeding the average vessel length (Tixier et al., 2013) by three times to reduce artifacts from vessel length disparities (Zimmermann and Jeje, 1981). Compared to Tixier et al. (2013), both these factors resulted in 25% greater discrepancies between *K*_h_ and *K*_ht_ in basal stem segments after the identification of functional vessels by dye perfusion.

### Choice of tracer

Various tracers able to pass through pit membranes were previously utilized for the visualization of conductive elements of xylem. Selectively lignin-binding dye solutions such as safranin, fuchsine, and toluidine blue were mostly preferred for the visualization of conductive elements across woody and herbaceous plant species (Supplemental Table 1). However, all of these dyes frequently caused the undesirable staining of cells (fibers, in particular) surrounding the conductive elements (Čermák et al., 2002; Sano et al., 2005; Halis et al., 2012). Although the xylem of *Arabidopsis* is formed by a smaller proportion of fibers than in woody plant species, their intensive staining in the PX area precludes the easy identification of PX vessels. The high diffusion rate and poor contrast between functional and non-functional vessels excludes the use of these dyes for vessel identification in *Arabidopsis thaliana* (Figures 1G–I).

The strong fluorescence of FB28 and its ability to selectively bind to cellulose enabled the precise determination of secondary cell walls and thus helped to identify and select individual functional vessels (Supplemental Figure 1E). However, differentiation of the autofluorescence signal (the fluorescence of lignin, cellulose and hemicellulose in secondary cell walls, in particular) from the signal of the tracer was necessary in fluorescence observations. Although the excitation and emission spectra of bound FB28 dye, lignin, cellulose, and hemicellulose overlap (Hon, 2001), the chosen 0.3% concentration of the FB28 solution ensured contrastive staining of secondary cell walls without the detection of any autofluorescence after the proper setting of the exposure time.

Because the dye solution is colorless, additional observation of the same intact cross section can easily be made in BF. On the other hand, there are limitations to the histochemical staining (e.g., with phloroglucinol or toluidine blue) of cross sections perfused with FB28 dye due to quenching of the FB28 fluorescence signal.

### Dye application

The method of dye application can significantly contribute to staining quality. Transpiration is commonly used to pull a dye solution through conductive pathways of living trees in particular (Sakamoto and Sano, 2000; Orians et al., 2004; Sano et al., 2005; Umebayashi et al., 2007, 2010). However, the limited mobility of a tracer (Sano et al., 2005) and therefore the long time needed for confident staining (several hours) excludes such application in *Arabidopsis*.

In the case of thin stems or branches, dye application techniques are commonly based on the perfusion of segments at an overpressure gradient of about 100 kPa (Zanne et al., 2006) or on pulling the solution at a pressure gradient generated by a vacuum pump (Tang and Boyer, 2002; Hacke et al., 2006; Halis et al., 2012, 2013; Barnard et al., 2013). Although both techniques ensure perfusion of the dye solution through all conductive vessels in a short time, we found, in the case of *Arabidopsis* inflorescence stems, that dye perfusion at overpressure resulted in the significant presence of dye in other than conductive tissues (Figure not shown). In contrast to classical dyes, perfusion of the colorless FB28 dye through the stem segment was monitored accurately according to the amount of pulled solution. The combination of a pump equipped with a suction regulator and a precise analytical balance enabled the flow rate of the solution to be regulated and, thus, kept stable.

### Implications of functional vessel identification

The proper identification of conductive vessels by the technique described above significantly improves the results of hydraulic and anatomical analyses. Our results clearly show that vessels with fully developed secondary cell walls mistakenly identified as conductive in cross sections form a significant proportion of the total vessel count. It is obvious that mistakes in the assumed number of functional vessels can lead to significant discrepancies in quantitative anatomical analyses.

The anatomical characteristics of vessels (e.g., diameter) are preferably calculated from cross sections not perfused with a tracer (Zanne et al., 2006; Voelker et al., 2011) compared to sections with stained conductive elements (Cai and Tyree, 2010). In the case of staining with fluorescent dye, the analysis of parameters using EF images can lead to some differences in the data. As already shown, inner vessel diameters calculated from EF images were significantly greater by 0.23 μm compared to identical vessels in BF images.

Vessel diameters are used for the calculation of theoretical hydraulic conductivity *K*_ht_ based on the Hagen-Poiseuille equation. Although conductivity increases with the fourth power of vessel diameter, the decline in the *K*_ht_ value in basal segments (as a consequence of the identification of an accurate number of functional vessels) was more than 10 times greater than the increase in *K*_ht_ (as a consequence of the 0.23 μm increase in measured diameters). In apical segments, the corresponding difference was only three times greater, emphasizing the greater importance of precise diameter evaluation in mutant lines with a lower number of vessels.

Comparisons of *K*_ht_ with experimentally measured *K*_h_ typically indicate great differences, as presented in many previous studies (Martre et al., 2000; Gloser et al., 2011; Jupa et al., 2013). Factors such as the resistivity of vessel end walls (Sperry et al., 2005) or perforation plates (Christman and Sperry, 2010), deviations from the shape of the ideal capillary, the variation in vessel diameter, or the incorrect determination of conductive vessels contribute most to the total difference. Nevertheless, the contribution of individual factors to the total difference between *K*_h_ and *K*_ht_ varies among plant species. Our data show that only the incorrect determination of conductive vessels in WT contributed to the difference—by 6.87% in the apical part and 18.88% in the basal part—and had a significant influence on the improvement of *K*_ht_ estimation (Figure 2).

Similarly, our results showed variability in the relative comparison of *K*_h_ with *K*_ht_. Although the methodology of *K*_h_ measurement and the various sizes and ages of plant samples in the same developmental stage contribute to the variation (data not shown), we consider the determination of conductive vessels as the most significant source of the variability. The correct determination of conductive vessels by dye perfusion reduced the coefficient of variation by 0.18. Simultaneously, the increase in *R*^2^ of 0.1412 reveals a stronger linear correlation between *K*_h_ and *K*_ht_ after dye perfusion.

The presented results clearly show significant improvements in the estimation of hydraulic conductivity, vessel number, and vessel diameter, as well as data correlation after the identification of conductive xylem elements. This suggests that precise identification of conductive vessels should be performed as an inseparable part of hydraulic and anatomical analyses of xylem, particularly in species with a low number of vessels such as *Arabidopsis*.

### Conflict of interest statement

The authors declare that the research was conducted in the absence of any commercial or financial relationships that could be construed as a potential conflict of interest.

## Abbreviations

BF: bright field microscopy
CLSM: confocal laser scanning microscopy
EF: epifluorescence microscopy
FB28: Fluorescent Brightener 28
PX: protoxylem
MX: metaxylem
SX: secondary xylem
*K*_h_: experimental hydraulic conductivity
*K*_ht_: theoretical hydraulic conductivity
*K*_s_: specific conductivity
*K*_hs_: experimental specific conductivity
*K*_hts_: theoretical specific conductivity.
