# Supplementary material for: An improved method for the visualization of conductive vessels in Arabidopsis thaliana inflorescence stems
Source: Front Plant Sci. 2015 Apr 9;6:211. doi: 10.3389/fpls.2015.00211 (PMC4391271; doi:10.3389/fpls.2015.00211)
Supplement: Supplementary file 1 [file Table1.PDF]

Supplementary material

An improved method for the visualization of conductive vessels in *Arabidopsis thaliana* inflorescence stems

Radek Jupa[1], Vojtěch Didi[2], Jan Hejátko[2], Vít Gloser[1*]

[1] Department of Experimental Biology, Faculty of Science, Masaryk University, Brno, Czech Republic
[2] Functional Genomics and Proteomics of Plants, Central European Institute of Technology, Masaryk University, Brno, Czech Republic

***Correspondence:** Vít Gloser, Department of Experimental Biology, Faculty of Science, Masaryk University, Kotlářská 2, 611 37 Brno, Czech Republic
VitGloser@sci.muni.cz

1. Tables

Table 1. Examples of studies since 1990 in which dye solutions were used as tracers to visualize xylem conductive pathways. SR – sulphorhodamine, S – safranin, BF – basic fuchsine, AF – acid fuchsine, F – fluorescein, AB – alcian blue, TB – toluidine blue, FG – fast green, CV – crystal violet.

**Study**	**Year**	**Dye**	**Concentration (%)**	**Studied plant species**
Canny	1990	SR	-	*Zea mays, Lupinus polyphyllus, Populus balsamifera, Mimosa sp., Polypodium virginianum, Pilea microphylla, Ficus diversifolia, Triticum aestivum*
Sperry and Tyree	1990	S	-	*Abies balsamea, Picea rubens, Juniperus virginiana*
Varney and Canny	1993	SR	0.5 mmol l	*Zea mays*
Shani *et al.*	1993	BF	-	*Vitis vinifera*

Granier *et al.*	1994	BF, S	0.005	*Quercus petraea, Q. robur*
Schurr and Schulze	1996	AF	-	*Ricinus communis*
Matsumura *et al.*	1998	TB, F	0.5, 0.01	*Pinus radiata*
Zwieniecki and Holbrook	1998	BF, AB	0.1	*Fraxinus americana, Acer rubrum, Picea rubens*
Tyree *et al.*	1999	FG	1.0	*Laurus nobilis*
Shane *et al.*	2000a	TB	0.005	*Zea mays*
Shane *et al.*	2000b	BF, SR	0.05, 0.05	*Zea mays*
Sakamoto and Sano	2000	S	0.2	*Salix sachalinensis*
Zwieniecki	2001	S	-	*Fraxinus americana*
Tang and Boyer	2002	S	0.07	*Zea mays*
Čermák *et al.*	2002	AF	1.0	*Laurus azorica*
Orians *et al.*	2002	SR	0.25	*Lycopersicon esculentum*
Schulte and Brooks	2003	S	0.5	*Pinus ponderosa, Pseudotsuga menziesii*
Orians *et al.*	2004	S	0.5	*Acer sacharum, Betula lenta, B. papyrifera, Populus tremuloides*
Maton and Gartner	2005	AF	1.0	*Larix occidentalis, Pinus flexilis, Picea abies, Sequoia sempervirens, Tsuga heterophylla, Calocedrus decurrens, Chamaecyparis nootkatensis, Juniperus virginiana, Ginkgo biloba, Abies concolor, Cedrus deodora, Picea pungens*
Sano *et al.*	2005	AF, S	0.5	*Populus sieboldii*

Orians *et al.*	2005	S	0.5	*Acer sacharum, Betula papyrifera, Liriodendron tulipifera, Castanea dentata, Fraxinus americana, Quercus rubra*
Hacke *et al.*	2006	S	0.05	*Carya glabra, Fraxinus pennsylvanica, Morus alba, Quercus gambelii, Q. prinus, Rhus trilobata, Acer grandidentatum, Arctostaphylos patula, Ceanothus velutinus, Larrea tridentata, Oxydendron arboreum, Paxistima myrsinites, Pueraria montana*
Zanne *et al.*	2006	TB	0.1	*Acer pensylvanicum, Ailanthus altissima, Betula populifolia, Buckleya distichophylla, Celtis occidentalis, Ehretia acuminata, Gleditsia aquatica, Hamamelis mollis, Ilex opaca, Kalopanax septemlobus, Liquidambar acalycina, Magnolia fraseri, Nyssa aquatica, Oxydendrum arboreum, Paulownia tomentosa, Picrasma quassioides, Platanus occidentalis, Sassafras albidum*
Keller *et al.*	2006	BF	0.1	*Vitis vinifera*
Chatelet *et al.*	2006	TB	0.1	*Vitis vinifera*
Ellmore *et al.*	2006	S	0.1, 0.5	*Acer sacharum, Betula papyrifera, Quercus rubra*
Jacobsen *et al.*	2007	CV, BF	0.1	*Malosma laurina, Rhus integrifolia, R. ovata, R. trilobita, Arctostaphylos glandulosa, A. glauca, Comarostaphylis diversifolia, Quercus agrifolia, Q. berberidifolia, Q. wislizeni, Garrya veatchii, Umbellularia californica, Ceanothus crassifolius, C. cuneatus, C. leucodermis, C. megacarpus, C. oliganthus, C. spinosus, Rhamnus californica, R. crocea, R. ilicifolia, Adenostoma fasciculatum, A. sparsifolium, Cercocarpus betuloides, Heteromeles arbutifolia, Prunus ilicifolia*
Umebayashi *et al.*	2007	AF	0.05, 0.1, 0.2, 0.5	*Pieris japonica*
Taneda and Tateno	2007	S	0.5	*Pueraria lobata*

Umebayashi *et al.*	2010	AF	0.2	*Dendropanax trifidus, Elaeagnus pungens, Aucuba japonica, Camellia japonica, C. sinensis, Daphniphyllum teijsmannii, Distylium racemosum, Eurya japonica, Ilex chinensis, I. crenata, I. pedunculosa, Illicium anisatum, Ligustrum japonicum, Litsea coreana, Machilus japonica, M. thunbergii, Myrica rubra, Myrsine seguinii, Neolitsea sericea, Pieris japonica, Pittosporum tobira, Rhaphiolepis indica, Symplocos lucida, S. myrtacea, Ternstroemia gymnanthera, Vaccinium bracteatum, Castanopsis sieboldii, Lithocarpus edulis, Quercus glauca, Q. myrsinaefolia, Q. salicina, Trochodendron aralioides*
Cai and Tyree	2010	BF	0.1	*Populus tremuloides*
Sano *et al.*	2011	AF	0.5	*Acer pictum, A. pseudoplatanus, Betula japonica, Cercidiphyllum japonicum, Fagus crenata, Fraxinus mandshurica, Ilex aquifolium, Juglans mandschurica, Kalopanax septembolus, Prunus sargentii, Quercus crispula, Q. robur, Salix sachalinensis, Sorbus commixta, Ulmus minor*
Voelker *et al.*	2011	AF	0.2	*Populus tremula* × *Populus alba*
Halis *et al.*	2012	TB	1.0	*Vitis vinifera*
Jacobsen *et al.*	2012	CV	0.1	*Vitis vinifera*
Plavcová and Hacke	2012	S	-	*Populus trichocarpa* × *Populus deltoides*
Halis *et al.*	2013	TB	1.0	*Calligonum comosum, Genista saharae, Limoniastrum guyonianum, Nitraria retusa, Retama retam, Tamarix gallica, Zizyphus lotus*
Barnard *et al.*	2013	AF	0.2	*Pinus contorta, P. ponderosa, Pseudotsuga menziesii*

2. References

Barnard, D. M., Lachenbruch, B., McCulloh, K. A., Kitin, P., and Meinzer, F. C. (2013). Do ray cells provide a pathway for radial water movement in the stems of conifer trees? *American Journal of Botany* 100, 322-331. doi: 10.3732/ajb.1200333

Cai, J., and Tyree, M. T. (2010). The impact of vessel size on vulnerability curves: data and models for within-species variability in saplings of aspen, *Populus tremuloides* Michx. *Plant, Cell and Environment* 33, 1059-1069. doi: 10.1111/j.1365-3040.2010.02127.x

Canny, M. J. (1990). What becomes of the transpiration stream? *New Phytologist* 114, 341-368. doi: 10.1111/j.1469-8137.1990.tb00404.x

Čermák, J., Jimenez, M. S., González-Rodríguez, A. M., and Morales, D. (2002). Laurel forests in Tenerife, Canary Islands. II. Efficiency of the water conducting system in *Laurus azorica* trees. *Trees-Structure and Function* 16, 538-546. doi: 10.1007/s00468-002-0198-y

Chatelet, D. S., Matthews, M. A., and Rost, T. L. (2006). Xylem structure and connectivity in grapevine (*Vitis vinifera*) shoots provides a passive mechanism for the spread of bacteria in grape plants. *Annals of Botany* 98, 483-494. doi: 10.1093/aob/mcl124

Ellmore, G. S., Zanne, A. E., and Orians, C. M. (2006). Comparative sectoriality in temperate hardwoods: hydraulics and xylem anatomy. *Botanical Journal of the Linnean Society* 150, 61-71. doi: 10.1111/j.1095-8339.2006.00510.x

Granier, A., Anfodillo, T., Sabatti, M., Cochard, H., Dreyer, E., Tomasi, M., Valentini, R., and Bréda, N. (1994). Axial and radial water flow in the trunks of oak trees – a quantitative and qualitative analysis. *Tree Physiology* 14, 1383-1396. doi: 10.1093/treephys/14.12.1383

Hacke, U. G., Sperry, J. S., Wheeler, J. K., and Castro, L. (2006). Scaling of angiosperm xylem structure with safety and efficiency. *Tree Physiology* 26, 689-701. doi: 10.1093/treephys/26.6.689

Halis, Y., Djehichi, S., and Senoussi, M. M. (2012). Vessel development and the importance of lateral flow in water transport within developing bundles of current-year shoots of grapevine (*Vitis vinifera* L.). *Trees-Structure and Function* 26, 705-714. doi: 10.1007/s00468-011-0637-8

Halis, Y., Mayouf, R., Benhaddya, M. L., and Belhamra, M. (2013). Intervessel connectivity and relationship with patterns of lateral water exchange within and between xylem sectors in seven xeric shrubs from the great Sahara desert. *Journal of Plant Research* 126, 223-231. doi: 10.1007/s10265-012-0514-6

Jacobsen, A. L., and Pratt, R. B. (2012). No evidence for an open vessel effect in centrifuge based vulnerability curves of a long-vesselled liana (*Vitis vinifera*). *New Phytologist* 194, 982-990. doi: 10.1111/j.1469-8137.2012.04118.x

Jacobsen, A. L., Pratt, R. B., Ewers, F. W., and Davis, S. D. (2007). Cavitation resistance among twenty-six chaparral species of southern California. *Ecological Monographs* 77, 99–115. doi: 10.1890/05-1879

Keller, M., Smith, J. P., and Bondada, B. R. (2006). Ripening grape berries remain hydraulically connected to the shoot. *Journal of Experimental Botany* 57, 2577-2587. doi: 10.1093/jxb/erl020

Maton, C., and Gartner, B. L. (2005). Do gymnosperm needles pull water through the xylem produced in the same year as the needle? *American Journal of Botany* 92, 123-131. doi: 10.3732/ajb.92.1.123

Matsumura, J., Booker, R. E., Donaldson, L. A., and Ridoutt, B. G. (1998). Impregnation of radiata pine wood by vacuum treatment: Identification of flow paths using fluorescent dye and confocal microscopy. *Iawa Journal* 19, 25-33.

Orians, C. M., Ardón, M., and Mohammad, B. A. (2002). Vascular architecture and patchy nutrient availability generate within plant heterogeneity in plant traits important to herbivores. *American Journal of Botany* 89, 270-278. doi: 10.3732/ajb.89.2.270

Orians, C. M., Smith, S. D. P., and Sack, L. (2005). How are leaves plumbed inside a branch? Differences in leaf-to-leaf hydraulic sectoriality among six temperate tree species. *Journal of Experimental Botany* 56, 2267-2273. doi: 10.1093/jxb/eri233

Orians, C. M., van Vuuren, M. M. I., Harris, N. L., Babst, B. A., and Ellmore, G. S. (2004). Differential sectoriality in long-distance transport in temperate tree species: evidence from dye flow, N-15 transport, and vessel element pitting. *Trees-Structure and Function* 18, 501-509. doi: 10.1007/s00468-004-0326-y

Plavcová, L., and Hacke, U. G. (2012). Phenotypic and developmental plasticity of xylem in hybrid poplar saplings subjected to experimental drought, nitrogen fertilization, and shading. *Journal of Experimental Botany* 63, 6481-6491. doi: 10.1093/jxb/ers303

Sakamoto, Y., and Sano, Y. (2000). Inhibition of water conductivity caused by watermark disease in *Salix sachalinensis*. *Iawa Journal* 21, 49-60. doi: 10.1163/22941932-90000236

Sano, Y., Morris, H., Shimada, H., De Craene, L. P. R., and Jansen, S. (2011). Anatomical features associated with water transport in imperforate tracheary elements of vessel-bearing angiosperms. *Annals of Botany* 107, 953-964. doi: 10.1093/aob/mcr042

Sano, Y., Okamura, Y., and Utsumi, Y. (2005). Visualizing water-conduction pathways of living trees: selection of dyes and tissue preparation methods. *Tree Physiology* 25, 269-275. doi: 10.1093/treephys/25.3.269

Schulte, P. J., and Brooks, J. R. (2003). Branch junctions and the flow of water through xylem in Douglas-fir and ponderosa pine stems. *Journal of Experimental Botany* 54, 1597-1605. doi: 10.1093/jxb/erg169

Schurr, U., and Schulze, E. D. (1996). Effects of drought on nutrient and ABA transport in *Ricinus communis*. *Plant, Cell Environment* 19, 665-674. doi: 10.1111/j.1365-3040.1996.tb00401.x

Shane, M. W., McCully, M. E., and Canny, M. J. (2000a). Architecture of branch-root junctions in maize: Structure of the connecting xylem and the porosity of pit membranes. *Annals of Botany* 85, 613-624. doi: 10.1006/anbo.2000.1113

Shane, M. W., McCully, M. E., and Canny, M. J. (2000b). The vascular system of maize stems revisited: Implications for water transport and xylem safety. *Annals of Botany* 86, 245-258. doi: 10.1006/anbo.2000.1171

Shani, U., Waisel, Y., Eshel, A., Xue, S., and Ziv, G. (1993). Responses to salinity of grapevine plants with split root systems. *New Phytologist* 124, 695-701. doi: 10.1111/j.1469-8137.1993.tb03860.x

Sperry, J. S., and Tyree, M. T. (1990). Water-stress-induced xylem embolism in three species of conifers. *Plant, Cell and Environment* 13, 427-436. doi: 10.1111/j.1365-3040.1990.tb01319.x

Taneda, H., and Tateno, M. (2007). Effects of transverse movement of water in xylem on patterns of water transport within current-year shoots of kudzu vine, *Pueraria lobata*. *Functional Ecology* 21, 226-234. doi: 10.1111/j.1365-2435.2006.01239.x

Tang, A. C., and Boyer, J. S. (2002). Growth-induced water potentials and the growth of maize leaves. *Journal of Experimental Botany* 53, 489-503. doi: 10.1093/jexbot/53.368.489

Tyree, M. T., Salleo, S., Nardini, A., Lo Gullo, M. A., and Mosca, R. (1999). Refilling of embolized vessels in young stems of laurel. Do we need a new paradigm? *Plant Physiology* 120, 11-21. doi: 10.1104/pp.120.1.11

Umebayashi, T., Utsumi, Y., Koga, S., Inoue, S., Matsumura, J., Oda, K., Fujikawa, S., Arakawa, K., and Otsuki, K. (2010). Xylem water-conducting patterns of 34 broadleaved evergreen trees in southern Japan. *Trees-Structure and Function* 24, 571-583. doi: 10.1007/s00468-010-0428-7

Umebayashi, T., Utsumi, Y., Koga, S., Inoue, S., Shiiba, Y., Arakawa, K., Matsumura, J., and Oda, K. (2007). Optimal conditions for visualizing water-conducting pathways in a living tree by the dye injection method. *Tree Physiology* 27, 993-999. doi: 10.1093/treephys/27.7.993

Varney, G. T., and Canny, M. J. (1993). Rates of water uptake into the mature root system of maize plants. *New Phytologist* 123, 775-786. doi: 10.1111/j.1469-8137.1993.tb03789.x

Voelker, S. L., Lachenbruch, B., Meinzer, F. C., Kitin, P., and Strauss, S. H. (2011). Transgenic poplars with reduced lignin show impaired xylem conductivity, growth efficiency and survival. *Plant, Cell and Environment* 34, 655-668. doi: 10.1111/j.1365-3040.2010.02270.x

Zanne, A. E., Sweeney, K., Sharma, M., and Orians, C. M. (2006). Patterns and consequences of differential vascular sectoriality in 18 temperate tree and shrub species. *Functional Ecology* 20, 200-206. doi: 10.1111/j.1365-2435.2006.01101.x

Zwieniecki, M. A., and Holbrook, N. M. (1998). Diurnal variation in xylem hydraulic conductivity in white ash (*Fraxinus americana* L.), red maple (*Acer rubrum* L.) and red spruce (*Picea rubens* Sarg.). *Plant Cell and Environment* 21, 1173-1180. doi: 10.1046/j.1365-3040.1998.00342.x

Zwieniecki, M. A., Melcher, P. J., and Holbrook, N. M. (2001). Hydraulic properties of individual xylem vessels of *Fraxinus americana*. *Journal of Experimental Botany* 52, 257-264. doi: 10.1093/jexbot/52.355.257
