# Supplementary material for: An improved method for the visualization of conductive vessels in Arabidopsis thaliana inflorescence stems
Source: Front Plant Sci. 2015 Apr 9;6:211. doi: 10.3389/fpls.2015.00211 (PMC4391271; doi:10.3389/fpls.2015.00211)
Supplement: Supplementary file 2 [file Image1.PDF]

# *Supplementary material*

# An improved method for the visualization of conductive vessels in *Arabidopsis thaliana* inflorescence stems

**Radek Jupa[1], Vojtěch Didi[2], Jan Hejátko[2], Vít Gloser[1*]**

[1] Department of Experimental Biology, Faculty of Science, Masaryk University, Brno, Czech Republic
[2] Functional Genomics and Proteomics of Plants, Central European Institute of Technology, Masaryk University, Brno, Czech Republic

***Correspondence:** Vít Gloser, Department of Experimental Biology, Faculty of Science, Masaryk University, Kotlářská 2, 611 37 Brno, Czech Republic
VitGloser@sci.muni.cz

## 2. Figures

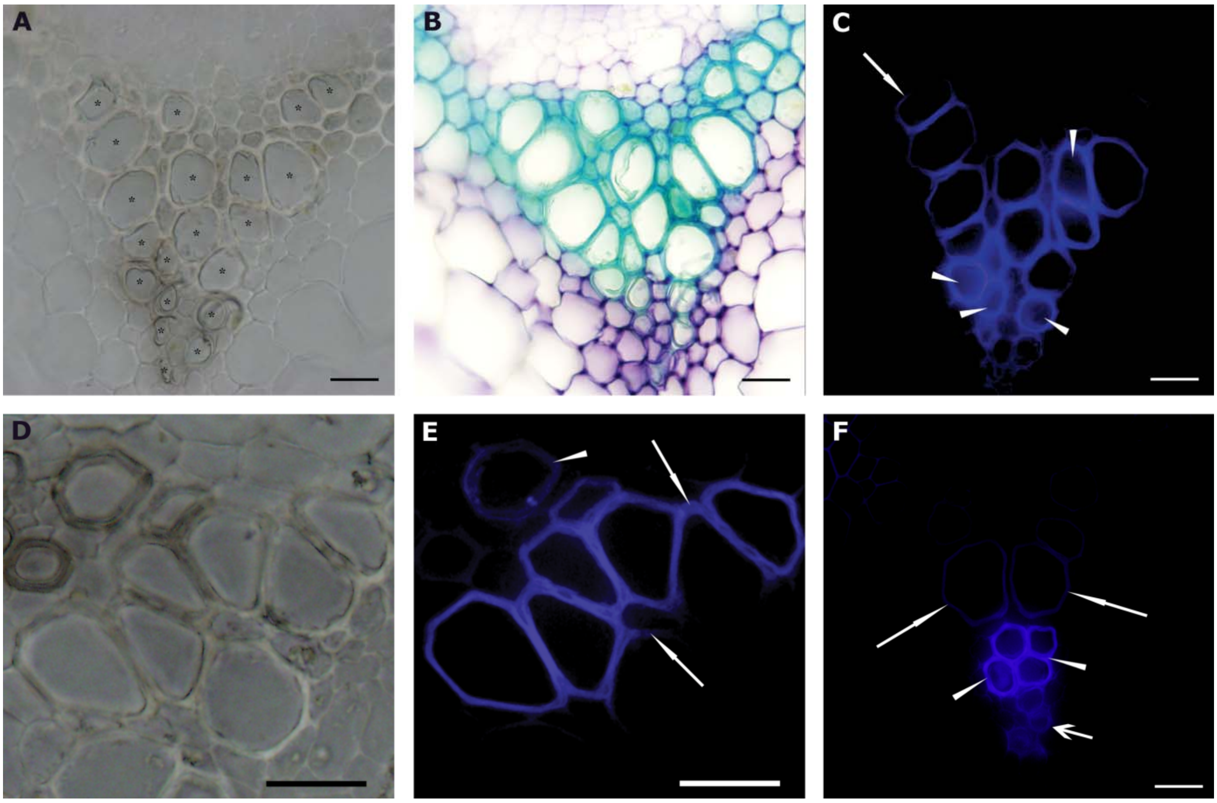


**Figure 1.** Native cross sections of individual vascular bundles in inflorescence stems of *Arabidopsis thaliana*. **(A)** Cross section of vascular bundle observed in bright field (BF). Asterisks show vessels with fully developed cell walls identified as conductive. **(B)** Transverse section histochemically stained with toluidine blue prepared sequentially in acropetal direction to the section in A. Toluidine blue stains lignified vessels in blue-green. **(C)** Identical cross section to A observed in epifluorescence (EF) after perfusion with Fluorescent Brightener 28 (FB28) dye solution. The arrow shows a metaxylem (MX) vessel identified as conductive with more than one half of the secondary cell wall perimeter stained. Arrowheads show vessels with remains of unbound dye resulting from their insufficient perfusion with water. **(D-E)** Detail of MX vessels perfused with FB28 and observed in BF **(D)** and EF **(E)**. The high contrast of secondary cell walls facilitates the identification of conductive vessels and simplifies selection of their lumen. Arrows show partially stained secondary cell walls of non-conductive vessels adjacent to conductive vessels. Arrowhead shows a protoxylem (PX) vessel of lower staining intensity. **(F)** Vascular bundle observed in EF after rough exposure time setting. Close arrows show the weak signal of non-conductive MX vessels, reduced after fine exposure setting. Arrowheads show the much stronger staining of conductive vessels and the open arrow shows the weaker staining of less conductive PX vessels. Scale bars 20 µm.
