# Supplementary material for: An improved method for the visualization of conductive vessels in Arabidopsis thaliana inflorescence stems
Source: Front Plant Sci. 2015 Apr 9;6:211. doi: 10.3389/fpls.2015.00211 (PMC4391271; doi:10.3389/fpls.2015.00211)
Supplement: Supplementary file 3 [file Image2.PDF]

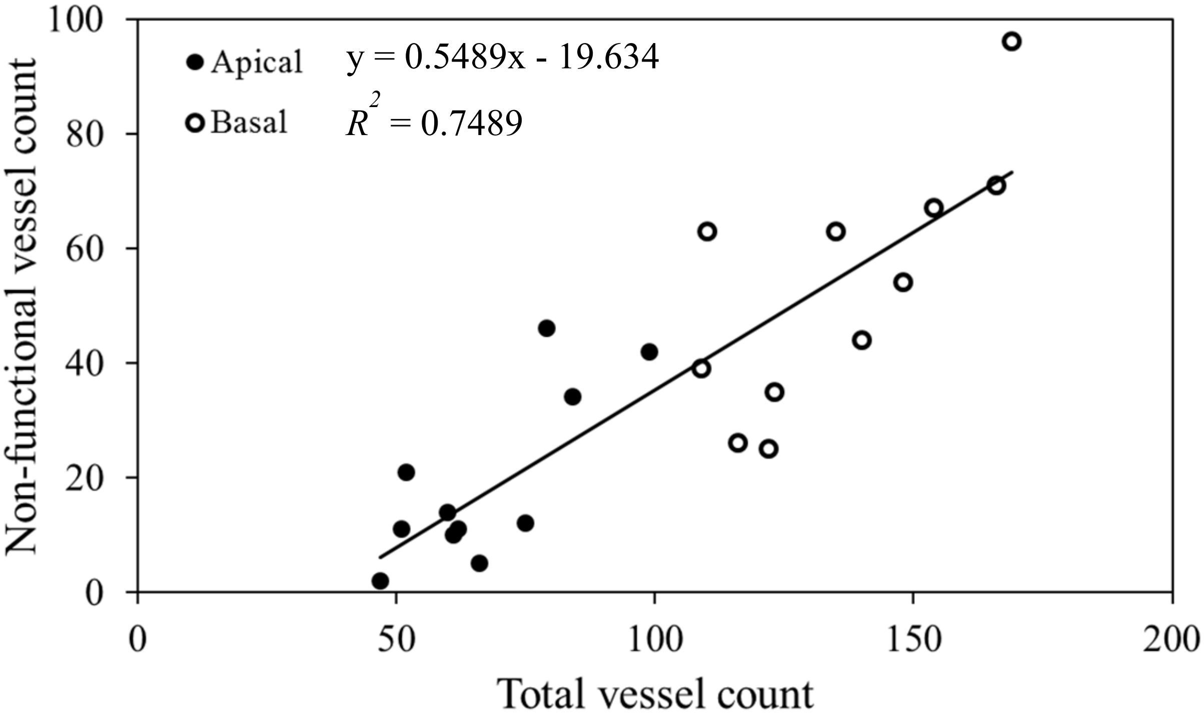

Figure 2. Relationship between total vessel count (i.e. vessels with fully developed secondary cell walls identified in cross sections observed in bright field) and the number of non-functional (non-conductive) vessels in eleven apical (closed circles) and basal (open circles) inflorescence stem segments of *Arabidopsis thaliana*. The number of non-functional vessels was calculated as the difference between total vessel count and the number of vessels identified after perfusion of the segments with Fluorescent Brightener 28 dye solution. Data were significantly correlated ($P < 0.001$) and fitted by linear regression ($R^2 = 0.7489$).
